# Case report: appendicitis induced *Staphylococcus aureus* and *Klebsiella pneumoniae* bacteremia in a young healthy male

**DOI:** 10.1186/s12245-021-00358-5

**Published:** 2021-07-19

**Authors:** Jan Arne Arne Deodatus, Sander Ferdinand Emiel Paas, Gerrit Hendrik Johan Wagenvoort, Marije Matilde de Kubber

**Affiliations:** Isala Hospital, Dokter van Heesweg 2, 8025 AB Zwolle, The Netherlands

**Keywords:** Appendicitis, Complicated appendicitis, Bacteremia, *Staphylococcus aureus*, *Klebsiella pneumoniae*, *Staphylococcus aureus* bacteremia

## Abstract

**Background:**

Appendicitis is one of the most frequently encountered conditions at the emergency department. Distinction is made between complicated and uncomplicated appendicitis. Complicated appendicitis may cause serious intra-abdominal infection, bacteremia, or sepsis. Emergency health providers should be highly alert to any early signs indicating such complications.

**Case presentation:**

We present the case of a healthy young male with a gangrenous appendicitis, who received antibiotics and underwent appendectomy. Blood cultures showed unequivocal *Staphylococcus aureus* and concomitant *Klebsiella pneumoniae* bacteremia requiring prolonged antibiotic treatment and further diagnostic evaluation.

**Conclusions:**

Although rare, appendicitis can cause *Staphylococcus aureus* and *Klebsiella pneumoniae* bacteremia with extensive implications for workup and antibiotic management. Our case stresses the importance of obtaining cultures in patients with suspicion of bacteremia given its consequences for clinical management.

## Background

Abdominal pain causes approximately 5% of emergency department (ED) visits, and appendicitis remains one of the major underlying causes [1]. The pooled incidence of appendicitis ranges from 100 to 151 per 100.000 in Western countries, and the lifetime risk is reported to be around 7% [2, 3]. Surgical appendectomy and empiric antibiotics remain the foundation of treatment, although more recently conservative (antibiotic) therapy of uncomplicated appendicitis has become a valid alternative [4]. Complicated appendicitis may cause serious intra-abdominal infection, bacteremia, or sepsis, requiring additional (usually antibiotic) management [5, 6].

Appendicitis is known to cause mixed aerobic and anaerobic infections, including *Escherichia coli*, *Streptococcus* spp., *Pseudomonas* spp., *Bacteroides* spp., and *Klebsiella* spp. However, presence of *Staphylococcus aureus* in appendiceal lumen, serosa, or intra-abdominal fluid is only reported in 0.68-3.7% of cases [7–9]. Safaya et al. presented the first known cases of methicillin-resistant *Staphylococcus aureus* (MRSA) positive peritoneal fluid cultures in acute appendicitis [10].

As far as we know, there are no current publications of *Staphyloccus aureus* bacteremia and few published cases of a *Klebsiella* bacteremia in acute appendicitis [8, 11]. We present a rare case of acute appendicitis complicated by a *Staphyloccus aureus* and concurrent *Klebsiella pneumoniae* bacteremia in a healthy young patient.

## Case presentation

A 29-year-old male patient presented to the ED with migrated right sided abdominal pain for 2 days. He reported rigors and fever up to 39 °C (measured orally at home), nausea, and vomiting, but no change in defecation. His medical history was unremarkable and he took no medication.

On physical examination, there were no signs of acute distress. He had a temperature of 37.2 °C, heart rate of 95 beats per minute, a blood pressure of 130/80 mmHg and saturation of 99% on room temperature without tachypnea. There was local tenderness in the right upper abdominal quadrant upon palpation without guarding. No jaundice was observed, and no abnormalities were found on auscultation of heart and lungs. Laboratory results showed elevated infection parameters (leukocyte count 11.1 × 10^9^/L, CRP 63 mg/L) and liver function tests (ASAT 208 U/L, ALAT 212 U/L, AF 50 U/L, GGT 93 U/L, total bilirubin 34 U/L, conjugated bilirubin 13 μmol/L). LDH was slightly elevated (305 U/L) and platelet count was slightly decreased (128 ×10^9^/L). The estimated glomerular filtration rate was normal (eGFR 80 mL/min, creatinine 108 μmol/L) with a BUN of 8.7 mmol/L. Urinalysis was positive for albumin and urobilinogen. Otherwise, laboratory results were normal. An abdominal ultrasound showed an enlarged appendix with fat-infiltration without signs of perforation. The patient was started on ceftriaxone (2000 mg once daily) and metronidazole (500 mg three times daily) after three sets (aerobic and anaerobic) of blood cultures were obtained. A laparoscopic appendectomy was performed on the same day, where a gangrenous appendix localized on the inferior liver border was found (possibly explaining the elevated liver function tests).

One day after admission, all six blood cultures were found positive for both *Klebsiella pneumoniae* and *Staphylococcus aureus* (*S. aureus*) susceptible for ceftriaxone. Thorough re-assessment and physical examination revealed no other infectious focus, no clinical stigmata of endocarditis, and no new cardiac murmurs. Transthoracic cardiac ultrasound (TTE) showed no signs of valvular vegetation. On the second day after surgery, the fever increased (38.6 °C) and the patient reported increasing general malaise. The antibiotic regime was switched to meropenem 1000 mg three times daily after control blood cultures were drawn in order to cover potential extended spectrum beta-lactamase (ESBL)-producing bacteria. After this, the fever subsided, and the clinical condition improved. An abdominal ultrasound was performed on the 4th day after surgery without signs of complications. The patient was discharged in good condition on the 8th day after admission with continuous intravenous flucloxacillin (12 g/24 h) until 14 days after the control blood cultures, which were all negative. By then, the patient had made a speedy recovery and all laboratory results had normalized.

## Discussion

Even though appendicitis is one of the most commonly encountered conditions at the ED, this case re-emphasizes how it may present with grave, unexpected infectious complications. We shall briefly review the involved pathogens and discuss current literature on the acquisition of cultures (blood and intra-abdominal) in the light of the presented case.

*Klebsiella pneumoniae* is a Gram-negative, facultative anaerobic bacterium that is found to colonize the skin, upper and lower gastrointestinal tracts. It is a well-known pathogen to cause upper respiratory tract infection, and is one of the major causative agents in gastro-intestinal tract infections [7–9, 11, 12]. An emerging problem from *Klebsiella pneumoniae* infection is the increasing number of resistant strains [12].

*Staphylococcus aureus* is a Gram-positive, facultative anaerobe that is commonly found as commensal bacterium of the skin and upper respiratory tract. Fecal carriage is reported to be 26% (CI 16.8-36.3%), but it is seldomly found in appendiceal cultures [7–9, 13]. Despite its frequently commensal relationship, *staphylococcus aureus* may rapidly become pathogenic and cause serious skin infections and pneumonia, as well as life-threatening bacteremia (SAB) [14]. We are unaware of any reports of an appendicitis-induced SAB.

SAB is a serious condition with an all-cause 30 day mortality of 10-30%, resulting in 2 to 10 deaths per 100,000 annually (higher mortality rates have been reported in MRSA bacteremia) [15]. Patients with SAB are prone to an array of complications, most notably from biofilm formation and metastatic infection [16, 17]. These may lead to persisting device-related infections, endocarditis, spondylodiscitis, (epidural) abscess, and septic arthritis. Failure to recognize such complications early may lead to relapse bacteremia and more complications, necessitating long-term antibiotic treatment. When SAB is diagnosed, appropriate antibiotic treatment should be initiated immediately. Also, the patient should be thoroughly re-evaluated for other portals of entry and metastatic infection (including echocardiography) and control blood cultures should be taken 2-4 days after initial cultures [18].

In this case, a TTE was obtained and showed no signs of valvular vegetation. No additional transesophageal ultrasound (TEE) was performed given the recent and appropriately treated focus of infection. Thorough history and physical examination revealed no signs of endocarditis stigmata, new cardiac souffles, or other foci of infection. Since susceptibility patterns were available very soon after blood cultures became positive and showed good susceptibility to ceftriaxone, it was decided to continue the initiated scheme. However, when the patient seemed to deteriorate and have persistent fever on the second day after admission, this was switched to meropenem for empirical coverage against possible ESBL-producing bacteria (carriage in the Netherlands around 6%) [19]. The SAB was regarded as uncomplicated once control blood cultures remained negative; hence, the patients was treated with intravenous antibiotics for 14 days.

Given the severity of a SAB and increasing antibiotic resistance, our case re-emphasizes the importance of adequate (blood) culturing at the ED to guide further antibiotic treatment. The indication for acquiring blood cultures in suspected infection at the ED has been subject to debate. Several predictor tools have been published that may help to reduce unnecessary blood cultures while not missing true bacteremia [20–24]. The most promising and externally validated prediction tool, by Shapiro et al., consists of several minor (1 point) and major (2 to 3 points) criteria with a score of 2 or more pointing toward an indication for blood cultures (Table 1). The authors report a sensitivity of 0.97 (CI 0.94-1.00), specificity of 0.29 (CI 0.26-0.31) and area under the curve of the receiver operated characteristic curve (AUC) of 0.75. External validation studies reported a sensitivity of 0.94-0.95 and specificity of 0.27-0.48 [25].

**Table 1:** The Shapiro decision rule as presented in the original article. A score of ≥ 2 is an indication to obtain a blood culture. If the score is lower, a blood culture is not indicated by the rule. With courtesy to Shapiro et al. [25]

Major criteria	Minor criteria (1 point each)
Suspected endocarditis (3 points) Temperature > 39.3 **°**C (3 points) Indwelling vascular catheter (2 points)	Temperature 38.3-39.3 °C Age > 65 years Chills Vomiting Hypotension (SBP < 90 mmHg) White blood cell count > 18.000 cells/μL Bands > 5% Platelets < 150.000 cells/μL Creatinine > 176 μmol/L

In our patient, the indication for blood cultures was not obvious and may not have been performed in many ED’s: he was immunocompetent, had no fever (37.2 °C at time of presentation on the ED), and there were no clinical signs of acute distress. However, he would have scored 4 minor criteria in the Shapiro score (reported fever, chills and vomiting, and platelets < 150.000/μL^9^), advocating in favor of blood cultures. Additionally, leukocyte differentiation showed a leukocyte band count of 1.85 × 10^9^/L, but was only available after the patient had already been admitted and started on antibiotics. Eventually, these blood cultures had important implications for antibiotic management and helped to prevent septic complications. Hence, we advocate maintaining a low threshold for obtaining blood cultures at the ED, especially in the light of the increasing number of multidrug resistant bacteria [9]. Although the decision about obtaining blood cultures remains largely based on clinical gestalt, we feel that clinical prediction tools such as the Shapiro score may be helpful to guide blood-culturing at the ED.

Finally, although there were no intra-abdominal cultures performed, this case emphasizes that appendicular tissue cultures should be considered in complicated appendicitis. Historically, this was almost standard practice, but later publications doubt its efficacy. Some authors suggest that routine cultures should be abandoned as they do not aid treatment strategy, but most of these papers were regarded as having limited scientific quality in a review in by Davies et al. [5, 6, 26, 27]. As of now, it seems appropriate to consider cultures in the case of immune-compromised patients and in complicated appendicitis (gangrenous or perforated), as the number of (resistant) positive cultures is higher in these groups [28].

Although empiric antibiotic treatment is effective in most patients, early (blood or intra-abdominal) culturing may help guide antibiotic modification in abdominal infection. This is especially true in a nonresponsive patient and in the light of increased occurrence of multi-drug resistance bacterial strains [9]. Our case stresses that a low threshold for obtaining cultures should be maintained, even when there is a low suspicion of bacteremia. Whether to start empiric antibiotic treatment remains to be decided on a case-by-case basis and depends on local guidelines.

## Data Availability

Not applicable

## Abbreviations

SAB: *Staphylococcus aureus* bacteremia
ED: Emergency department
MRSA: Methicillin-resistant *Staphylococcus aureus*
ESBL: Extended spectrum beta-lactamase
TTE: Transthoracic ultrasound
TEE: Transesophageal ultrasound
