# Correction to: Case report: appendicitis induced *Staphylococcus aureus* and *Klebsiella pneumoniae* bacteremia in a young healthy male

**DOI:** 10.1186/s12245-021-00386-1

**Published:** 2021-11-01

**Authors:** Jan Arne Deodatus, Sander Ferdinand Emiel Paas, Gerrit Hendrik Johan Wagenvoort, Marije Matilde de Kubber

**Affiliations:** Isala Hospital, Dokter van Heesweg 2, 8025 AB Zwolle, The Netherlands

**Correction to: Int J Emerg Med 14, 36 (2021)**

**https://doi.org/10.1186/s12245-021-00358-5**

Following publication of the original article [1], the corresponding author reported that their middle name, Arne, was repeated in the authorship panel.

The correct authorship list is available in this Correction article and the author apologizes for the error.

The original article [1] has been corrected.
